# Independent and joint associations of skeletal muscle mass and physical performance with all-cause mortality among older adults: a 12-year prospective cohort study

**DOI:** 10.1186/s12877-022-03292-0

**Published:** 2022-07-18

**Authors:** Chia-Ing Li, Chiu-Shong Liu, Chih-Hsueh Lin, Shing-Yu Yang, Tsai-Chung Li, Cheng-Chieh Lin

**Affiliations:** 1 School of Medicine, College of Medicine, China Medical UniversityJingmao RdBeitun Dist, No. 100, Sec. 1, 406040 Taichung, Taiwan; 2 Department of Medical Research, China Medical University Hospital, Taichung, Taiwan; 3 Department of Family Medicine, China Medical University Hospital, Taichung, Taiwan; 4 Department of Public Health, College of Public Health, China Medical University, Taichung, Taiwan; 5 Department of Healthcare Administration, College of Medical and Health Science, Asia University, Taichung, Taiwan

**Keywords:** Skeletal muscle mass, Physical performance, All-cause mortality

## Abstract

**Background:**

Decreased skeletal muscle mass and low physical performance are independently associated with increased mortality in elderly individuals. However, little is known about the effects of skeletal muscle mass combined with physical performance on the prediction of mortality risk among community-dwelling older adults. This study aimed to determine the combined effects of skeletal muscle mass and physical performance on total mortality.

**Methods:**

A community-based prospective cohort study was conducted among 641 participants aged 65 and older in 2009. The height-adjusted skeletal muscle index (hSMI) and the weight-adjusted SMI (wSMI) were determined by dual-energy X-ray absorptiometry examination. Physical performance tests measured at baseline included gait speed (GS), timed up-and-go (TUG) test, timed chair stand (TCS), weight-adjusted leg press (WaLP), and handgrip strength (HS). Cox proportional hazards regression models were applied to determine the adjusted hazard ratios (HRs) of mortality with 95% confidence intervals (95% CIs) for baseline skeletal muscle mass, physical performance, and traditional risk factors.

**Results:**

During the follow-up of 12 years, 198 (30.89%) participants died. Low hSMI, low GS, high TUG, high TCS, low WaLP, and low HS were associated with high risks of mortality after the adjustment for confounders. The results of receiver operating characteristic (ROC) curve analyses revealed the values of ROC for models with additional consideration for TUG or all indicators significantly improved the discriminatory ability of mortality compared with the model with traditional factors (all *P* < 0.05). Elders with low hSMI and low GS (HRs = 4.33, 95% CI: 2.76–6.78), high TUG (4.11, 2.60–6.48), high TCS (2.97, 1.92–4.59), low WaLP (3.19, 2.13–4.79), and low HS (4.08, 2.70–6.17) were associated with high risks of mortality compared with those with high hSMI and their corresponding counterparts.

**Conclusion:**

The hSMI and physical performance are significantly associated with increased risks of all-cause mortality. The combined use of hSMI and physical performance can provide improved risk stratification, which may be appropriately used as a screening tool targeting high-risk elders for the effective prevention of sarcopenia-related mortality.

**Supplementary Information:**

The online version contains supplementary material available at 10.1186/s12877-022-03292-0.

## Background

Most countries in Asia are facing the problem of a rapidly aging population, especially in Taiwan [1]. As population ages, the incidence of skeletal muscle mass loss and physical performance limitation will increase. Age-related loss of skeletal muscle mass leads to a decline in physical performance, also referred to as sarcopenia. Sarcopenia has been considered one of the important geriatric syndromes [2, 3], and it is characterized by the global and progressive decrease in muscle mass and strength, severely limiting the physical performance; it is a major contributing factor to the increased risk of adverse health outcomes in elderly individuals, including physical disability, functional dependency, increased co-morbidity, and premature death [4–8]. A broad understanding of the effect of elderly skeletal muscle mass and physical performances on adverse outcomes and identification of elderly people at high risks of adverse outcomes have important implications for health care providers and policy makers in response to huge health care challenges.

Skeletal muscle plays a key role in metabolic function, contributing to glucose uptake and storage, and has associations with muscle strength and physical performance. Thus, an increase in metabolic need resulting in skeletal muscle loss may be associated with adverse health and mortality [9]. Skeletal muscle index (SMI) and physical performance are independently associated with mortality [10–29]. Observational evidence also has a close association between skeletal muscle mass index and physical performance [30–32]. Thus, several investigators became interested in whether skeletal muscle mass index and physical performance are independent predictors of mortality when both measures are considered [26, 28, 29]. These studies further indicated that physical performance (such as muscle strength, timed up-and-go (TUG), etc.) is a more important predictor than SMI [26, 28, 29]. However, they did not comprehensively consider the various domains of physical performance indicators. Thus, whether or to what extent SMI and physical performance are independently or jointly associated with all-cause mortality in elderly individuals remains unclear. Therefore, more evidence is required to explore the associations between SMI and physical performance with mortality.

We searched literature thoroughly regarding the effects of skeletal muscle mass and physical performance on mortality in older or elderly individuals and found several related studies [10–29, 33–35]. Among these studies, several explored the individual effect of skeletal muscle mass [10–15, 33, 34], others examined the individual effect of physical performance [16–23, 35]. Although several studies investigated the effect of both simultaneously [24–29], with research being conducted in Finland [24], Australia [27], USA [25, 26, 29], and Korea [28], they reported independent effects of measures for skeletal muscle mass or physical performance by comparing their discriminatory ability on predicting mortality [24, 25, 27–29] except one study reporting joint effect of low muscle mass and low muscle strength [26]. However, the definition of sarcopenia proposed by the European Working Group on Sarcopenia in Older People (EWGSOP) or Asian Working Group for Sarcopenia (AWGS) is a type of joint effect of skeletal muscle mass and physical performance. In addition, these studies did not comprehensively consider various domains of physical performance indicators but focused on one or two domains of physical performance, such as gait speed (GS) only [25], leg muscle strength only [26], leg muscle and grip strength [28, 29], and grip strength and TUG [27]. New scientific evidence regarding joint effect of muscle mass and physical performance must be added in this line of research question among older adults. The current study can provide information about the joint effects of skeletal muscle mass and multiple physical performance measures in predicting all-cause mortality.

## Method

### Study design and study subjects

We conducted a community-based prospective cohort study among all individuals aged 65 and over in Taichung City, Taiwan in 2009. A total of 3,997 elderly residents of the North District of Taichung City were recruited in 2009. All individuals were invited to participate in the study by letter, phone, and home visit. Exactly 2,750 subjects were considered eligible after assessment, and 1,347 eligible subjects accepted our invitation to participate with an overall response rate of 49.0%. After the exclusion of participants without complete data for baseline characteristics, skeletal muscle mass, and physical performance, 641 participants were included in the final analysis (Supplement Figure S1). This present study was approved by the Ethical Review Board of China Medical University Hospital (DMR 97-IRB-055 & CMUH108-REC2-161). Written informed consent was obtained from all the study participants. All methods were performed in accordance with the relevant guidelines and regulations.

### Measurements

#### Sociodemographic factors, lifestyle behaviors, and disease history

All subjects underwent a face-to-face interview with a standardized questionnaire to collect baseline characteristics consisting of socio-demographic characteristics, educational attainment level, marital status, smoking habits, habitual ethanol intake, regular exercise, personal history of hypertension, presence of diabetes, heart disease, stroke, and cancer. Persons who reported to have the characteristics of smoking, alcohol drinking, regular exercise and history of chronic diseases were placed into groups based on the specific characteristic. Those in the non-regular exercise group didn’t exercise regularly during the past month, whereas those in regular exercise group exercised regularly at least 2 times per week for 20 min during the past month. Among these participants, 146 frail or prefrail persons joined a randomized control trial, receiving home-based exercise instruction or supervised exercise program [36]. Participants receiving supervised exercise undertook a 3-month exercise training program under the supervision of a physical therapist. The participants undertook 3 exercise sessions per week, with a duration of 1.5 h per session, including 10-min warm-up and stretching activities, as well as 30-min aerobic exercise setting at between 70 and 85% of the predicted maximum heart rate, and resistance training (including strengthening of the elbow flexors utilizing dumb bells, hand grip muscles against grip strength trainers with different resistances, and lower limb muscles with a leg press machine). The participants receiving home-based exercise instruction attended a 15-min session of home-based exercise instructions. They were asked to exercise at least 3 times per week at home. Ten-page illustrated handouts describing calisthenics and resistance exercise for the upper and lower limbs were given.

#### Mini-mental State Examination (MMSE)

The MMSE was developed by Folstein M.F. and Folstein S.E. [37]. This questionnaire is designed for the graded evaluation of patients with cognitive impairment and now has become a widely used test to screen for cognitive disorders in epidemiological studies and follow-up cognitive changes in clinical trials. The MMSE contains items about time and place orientation, registration, attention and calculation, recall, and language and constructional abilities. The total score ranges between 0–30, and the cut-point score is variant with different educational levels. Cognitive impairment was considered if the total score of MMSE was less than 24, 21, and 16, with educational level of the participant more than 9 years, more than 6 years, and none, respectively [38].

#### Laboratory examination

Blood was drawn with minimal trauma from an antecubital vein in the morning after a 12 h overnight fasting and was sent for analysis within 4 h after blood collection. Blood tests, including fasting plasma glucose, total cholesterol, high-density lipoprotein-cholesterol, low-density lipoprotein-cholesterol, triglyceride, and creatinine, were analyzed by a biochemical autoanalyzer (Beckman Coluter, Lx-20, USA) at the Clinical Laboratory Department of China Medical University Hospital. The Chronic Kidney Disease Epidemiology Collaboration equation was used to calculate the estimated glomerular filtration rate [39].

#### SMI

We performed dual-energy X-ray absorptiometry (DXA) (Lunar DPX, General Electric, Madison, WI, USA) to determine the body composition of the subjects. The lean soft tissue mass and fat mass in the arms, legs, trunk, and entire body were determined using a DXA analysis software (Lunar enCORE; General Electric). The equipment was calibrated using a standardized employed each day. DXA is recommended by The European Working Group on Sarcopenia in Older People as the preferred alternative method to measure skeletal muscle mass for research and clinical use [2] because computer tomography and magnetic resonance imaging have the disadvantages of radiation exposure and/or high costs, which limit their use in routine clinical practice.

The height-adjusted SMI (hSMI) was derived by dividing the appendicular muscle mass (kg) by the square of height (m^2^), whereas the weight-adjusted SMI (wSMI) was calculated by dividing the appendicular muscle mass (kg) by weight (kg) [5].

#### Physical performance

Physical performance included measures of walking speed, TUG test, timed chair stands (TCS), weight-adjusted leg press (waLP), and handgrip strength (HS). The participants underwent all physical performance tests under the instructions of a physical therapists. For the walking test, the participants were asked to walk 5 m at their usual speed. The walking speed was calculated as 5 m divided by the recorded time. The TUG test required the participant to stand up from a sitting position, walk 3 m from that position, walk back to the chair, and sit down as immediately as possible, and the time elapsed was recorded [40]. The participants in the TCS test were asked to fold their arms across their chest, to sit firmly in a chair, and to stand up and sit down thrice, and the time elapsed was recorded [41]. The participants prohibited from using their hands for support during the test, and the test was repeated thrice. The shortest time elapsed was used for analysis. For waLP, submaximal leg press strength (a maximum of 10–15 repetitions) was measured by a leg press machine (AURA G3-S70, Matrix Fitness System, USA). Then, one-repetition maximum leg press strength was then estimated by the Brzycki formula [42]. WaLP was calculated by dividing the corresponding results by the weight of the participant. A dynamometer (TTM Dynamometer, Tsutsumi, Tokyo, Japan) was used to evaluate isometric HS in kg. Each hand was repeatedly measured thrice, and each hand’s average HS was calculated. The maximum average HS value of the left or right hand was defined as the participant’s HS.

#### Outcome ascertainment

The primary outcome measure was all-cause death status, which was determined through record linkage with the cause of death data in the Health and Welfare Data Science Center database. By linking National Registry of Death dataset, we obtained information on an individual’s date of death. The time of follow-up began with recruitment (index date) and ended with death or the end of follow-up (August 2021).

### Statistical analysis

In descriptive analysis, the baseline variables were shown as frequency (proportion) and assessed by the Chi-square tests for categorical variables and as means ± standard deviation (SD) and assessed by the t test for continuous variables. Unadjusted and adjusted Cox proportional hazards regression models were used to calculate hazard ratio (HR) and 95% confidence interval (CI) for mortality by sex-specific quartiles of hSMI, wSMI, and each physical performance measure (walking speed, TUG, TCS, waLP, and HS). The proportionality assumption was tested by including an interaction term for all exposure and outcome parameters. To test for the effect modification of sex, we separately included an interaction term between sex with each skeletal muscle index and physical performance measure in the multivariate Cox’s proportional hazards regression models. The joint contribution of skeletal muscle index and each physical performance measure was explored by entering the dummy variables of the combination of the aforementioned variables. The survival functions were estimated by the Kaplan–Meier method, and log-rank tests were used to determine the differences in entire survival functions among groups. The areas under the receiver operating characteristic curves (AUROCs) were calculated to evaluate the relative predictive ability of hSMI, wSMI, and each physical performance to correctly classify the elders’ mortality status; the nonparametric method was used to test the differences in the AUROCs among indexes of skeletal muscle mass and physical performance [43]. All analyses were performed with SAS version 9.4 (SAS, Cary, NC). All *p*-values were two-tailed, and a *p*-value < 0.05 was considered statistically significant.

## Results

During the median 12-year follow-up (interquartile range, 10.3–11.8 years), 198 (30.9%) elders died (of whom 135 (68.2%) from men). The mortality rate per 1000 person‐years was 39.13 for men and 19.13 for women. The distributions of baseline characteristics based on the mortality status are presented as frequency (proportion) or mean (SD) in Table 1. These results indicated that elders who died compared with those who did not, had higher prevalence of hypertension, diabetes mellitus, heart disease, stroke, cancer, and cognitive impairment, higher mean values of TUG and TCS, and lower mean values of GS and waLP.

**Table 1:** Comparisons of baseline socio-demographic factors, lifestyle behaviors, disease history, frailty status, cognitive impairment, biomarker, skeletal muscle index and physical performance according to mortality status (*n* = 641)

	Death N (%)		*P* value
Variables	No (*N* = 443)	Yes (*N* = 198)	
***Socio-demographic factors***			
Men	215 (48.53)	135 (68.18)	< 0.001
Age (years)			< 0.001
65–74	317 (71.56)	77 (38.89)	
75–84	122 (27.54)	97 (48.99)	
> 85	4 (0.90)	24 (12.12)	
Education			0.35
No education	46 (10.38)	14 (7.07)	
Primary education	118 (26.64)	59 (29.8)	
Secondary or tertiary education	279 (62.98)	125 (63.13)	
Married	332 (74.94)	145 (73.23)	0.72
***Lifestyle behaviors***			
Smoking	39 (8.80)	22 (11.11)	0.44
Alcohol drinking	63 (14.22)	31 (15.66)	0.72
Regular exercise	354 (79.91)	147 (74.24)	0.13
***Disease history***			
Hypertension	200 (45.15)	117 (59.09)	0.002
Diabetes Mellitus	49 (11.06)	42 (21.21)	0.001
Heart disease	110 (24.83)	79 (39.90)	< 0.001
Stroke	11 (2.48)	22 (11.11)	< 0.001
Cancer	18 (4.06)	17 (8.59)	0.03
***Cognitive impairment***	34 (7.67)	41 (20.71)	< 0.001
***Biomarker***			
Fasting plasma glucose (mg/dL)	106.75 ± 22.07	114.21 ± 31.3	0.003
Triglyceride (mg/dL)	115.65 ± 63.84	117.09 ± 72.81	0.81
High-density lipoprotein (mg/dL)	47.00 ± 13.38	45.03 ± 15.90	0.13
Low-density lipoprotein (mg/dL)	116.99 ± 31.38	109.65 ± 30.04	0.006
eGFR (mL/min per 1.73 m^2^)	77.11 ± 14.96	68.66 ± 18.79	< 0.001
***Height-adjusted SMI (kg/m***^***2***^***)***	6.96 ± 1.02	6.89 ± 1.04	0.39
***Weight-adjusted SMI (%)***	28.72 ± 4.17	29.07 ± 4.48	0.35
***Physical performance***			
Gait speed (m/s)	0.88 ± 0.18	0.73 ± 0.23	< 0.001
Timed up-and-go test (sec)	7.21 ± 2.04	9.31 ± 4.46	< 0.001
Timed chair stand (sec)	5.03 ± 1.48	6.25 ± 2.96	< 0.001
Weight-adjusted leg press (%)	96.87 ± 36.52	78.51 ± 36.95	< 0.001
Handgrip strength (kg)	28.00 ± 7.95	27.17 ± 7.73	0.22

Mean ± standard deviation for continuous variables; numbers (percentage) for categorical variables

*eGFR* Estimated glomerular filtration rate, *SMI* Skeletal muscle mass

Next, the bivariate associations between all-cause mortality and measures of skeletal muscle index and physical performance were assessed by the Kaplan–Meier survival curves for all-cause mortality based on quartiles of hSMI, GS, TUG, TCS, WaLP, and HS (Supplement Figure S2). The *P* values of log-rank test for all factors were < 0.001, except wSMI. The crude and multivariate-adjusted HRs with 95% CIs of all-cause mortality were estimated for quartiles of skeletal muscle index and physical performance (Table 2). Compared with subjects with hSMI, wSMI, GS, WaLP, and grip strength in the lowest quartile (quartile 1), those with hSMI, GS, WaLP, and grip strength in quartiles 2–4 had lower risks of mortality (crude HR = 0.52, 95% CIs = 0.36–0.75; 0.38, 0.26–0.57; 0.43, 0.29–0.63 for hSMI; 0.60, 0.41–0.88; 0.64, 0.44–0.94; and 0.57, 0.39–0.84 for wSMI; 0.37, 0.25–0.53; 0.28, 0.19–0.42; and 0.23, 0.15–0.35 for GS; 0.47, 0.33–0.67; 0.38, 0.26–0.55; and 0.25, 0.16–0.39 for WaLP, 0.53, 0.37–0.76; 0.34, 0.23–0.50; and 0.29, 0.19–0.44 for HS, respectively). Conversely, quartile 4 of TUG and quartiles 2–4 of TCS were associated with higher risks of mortality (HR: 3.97, 95% CI: 2.62–6.00 for TUG; 2.19, 1.31–3.66; 2.74, 1.66–4.50; and 4.68, 2.92–7.50 for TCS, respectively). After multivariate adjustment, hSMI, GS, TUG, TCS, WaLP, and HS were still independently associated with all-cause mortality.

**Table 2:** Hazards ratios of all-cause mortality for measures of skeletal muscle index and physical performance

Variables	N	Deaths	Person- years	Incidence rate	HR (95% CI)		
					Crude	Multivariate-adjusted^1^	Multivariate-adjusted^2^
***Height-adjusted SMI (kg/m***^***2***^***)***							
Q1 (Men 0.00–7.07; Women 0.00–5.69)	159	76	1487.59	51.09	1.00	1.00	1.00
Q2 (Men 7.08–7.58; Women 5.70–6.14)	160	46	1667.29	27.59	0.52 (0.36, 0.75)***	0.58 (0.40, 0.85)**	0.59 (0.40, 0.88)**
Q3 (Men7.59–8.05; Women 6.15–6.63)	161	36	1733.23	20.77	0.38 (0.26, 0.57)***	0.43 (0.29, 0.64)***	0.38 (0.25, 0.57)***
Q4 (Men ≥ 8.06; Women ≥ 6.64)	161	40	1735.44	23.05	0.43 (0.29, 0.63)***	0.56 (0.38, 0.83)**	0.52 (0.34, 0.78)**
***Weight-adjusted SMI (%)***							
Q1 (Men 0.00–28.97; Women 0.00–23.54)	159	67	1588.36	42.18	1.00	1.00	1.00
Q2 (Men 28.98–31.31; Women 23.55–25.58)	161	43	1661.54	25.88	0.60 (0.41, 0.88)**	0.72 (0.49, 1.07)	0.75 (0.50, 1.11)
Q3 (Men 31.32–33.61; Women 25.59–27.92)	163	47	1703.08	27.60	0.64 (0.44, 0.94)*	0.88 (0.60, 1.30)	0.96 (0.64, 1.43)
Q4 (Men ≥ 33.62; Women ≥ 27.93)	158	41	1670.57	24.54	0.57 (0.39, 0.84)**	0.71 (0.48, 1.07)	0.78 (0.51, 1.21)
***Physical performance***							
Gait speed (m/s)							
Q1 (Men 0.00–0.71; Women 0.00–0.68)	157	91	1403.52	64.84	1.00	1.00	1.00
Q2 (Men 0.72–0.85; Women 0.69–0.82)	155	43	1666.93	25.80	0.37 (0.25, 0.53)***	0.42 (0.28, 0.61)***	0.51 (0.34, 0.76)***
Q3 (Men 0.86–0.98; Women 0.83–0.92)	169	36	1806.21	19.93	0.28 (0.19, 0.42)***	0.38 (0.25, 0.59)***	0.50 (0.32, 0.78)**
Q4 (Men ≥ 0.99; Women ≥ 0.93)	160	28	1746.89	16.03	0.23 (0.15, 0.35)***	0.32 (0.20, 0.51)***	0.42 (0.26, 0.68)***
Timed up-and-go test (sec)							
Q1 (Men 0.00–6.05; Women 0.00–6.49)	161	30	1742.89	17.21	1.00	1.00	1.00
Q2 (Men 6.06–6.97; Women 6.50–7.21)	161	33	1737.21	19.00	1.10 (0.67, 1.81)	1.02 (0.62, 1.68)	1.06 (0.64, 1.75)
Q3 (Men 6.98–8.27; Women 7.22–8.67)	160	45	1715.24	26.24	1.53 (0.96, 2.43)	1.27 (0.79, 2.03)	1.24 (0.77, 2.01)
Q4 (Men ≥ 8.28; Women ≥ 8.68)	159	90	1428.21	63.02	3.97 (2.62, 6.00)***	2.66 (1.69, 4.21)***	2.06 (1.28, 3.30)**
Timed chair stand (sec)							
Q1 (Men 0.00–4.18; Women 0.00–4.29)	162	22	1810.18	12.15	1.00	1.00	1.00
Q2 (Men 4.19–4.92; Women 4.30–5.20)	160	44	1689.82	26.04	2.19 (1.31, 3.66)**	1.82 (1.09, 3.07)*	1.55 (0.92, 2.63)
Q3 (Men 4.93–5.89; Women 5.21–6.18)	160	52	1622.43	32.05	2.74 (1.66, 4.50)***	2.27 (1.37, 3.77)**	2.10 (1.25, 3.51)**
Q4 (Men ≥ 5.90; Women ≥ 6.19)	159	80	1501.12	53.29	4.68 (2.92, 7.50)***	3.14 (1.89, 5.19)***	2.49 (1.48, 4.18)***
Weight-adjusted leg press (%)							
Q1 (Men 0.00–73.18; Women 0.00–55.30)	161	84	1476.92	56.88	1.00	1.00	1.00
Q2 (Men 73.19–96.68; Women 55.31–77.59)	160	47	1654.5	28.41	0.47 (0.33, 0.67)***	0.52 (0.36, 0.76)***	0.56 (0.39, 0.81)**
Q3 (Men 96.69–123.45; Women 77.60–105.67)	159	39	1692.19	23.05	0.38 (0.26, 0.55)***	0.47 (0.32, 0.70)***	0.58 (0.39, 0.87)**
Q4 (Men ≥ 123.46; Women ≥ 105.68)	161	28	1799.95	15.56	0.25 (0.16, 0.39)***	0.37 (0.23, 0.57)***	0.48 (0.31, 0.77)**
Handgrip strength (kg)							
Q1 (Men 0.00–28.69; Women 0.00–18.79)	163	83	1497.71	55.42	1.00	1.00	1.00
Q2 (Men 28.70–32.99; Women 18.80–21.29)	157	50	1612.11	31.02	0.53 (0.37, 0.76)***	0.57 (0.40, 0.82)**	0.64 (0.44, 0.93)*
Q3 (Men 33.00–37.15; Women 21.30–24.26)	161	35	1756.11	19.93	0.34 (0.23, 0.50)***	0.40 (0.27, 0.60)***	0.42 (0.28, 0.63)***
Q4 (Men ≥ 37.16; Women ≥ 24.27)	160	30	1757.62	17.07	0.29 (0.19, 0.44)***	0.42 (0.27, 0.65)***	0.46 (0.29, 0.72)***

Multivariate adjustment^1^ for age, sex, education, marital status, smoking, alcohol drinking, physical activity, and exercising program

Multivariate adjustment^2^ for age, sex, education, marital status, smoking, alcohol drinking, physical activity, exercising program, hypertension, diabetes mellitus, heart disease, stroke, cancer, cognitive impairment, fasting plasma glucose, triglyceride, high-density lipoprotein, low-density lipoprotein, and estimated glomerular filtration rate

Incidence rate = (number of incident cases) / (1000 person-years); *SMI* skeletal muscle mass, *HR* Hazard ratio, *CI* Confidence interval

^*^:*p* < 0.05; **:*p* < 0.01; ***:*p* < 0.001

**Fig. 1:**
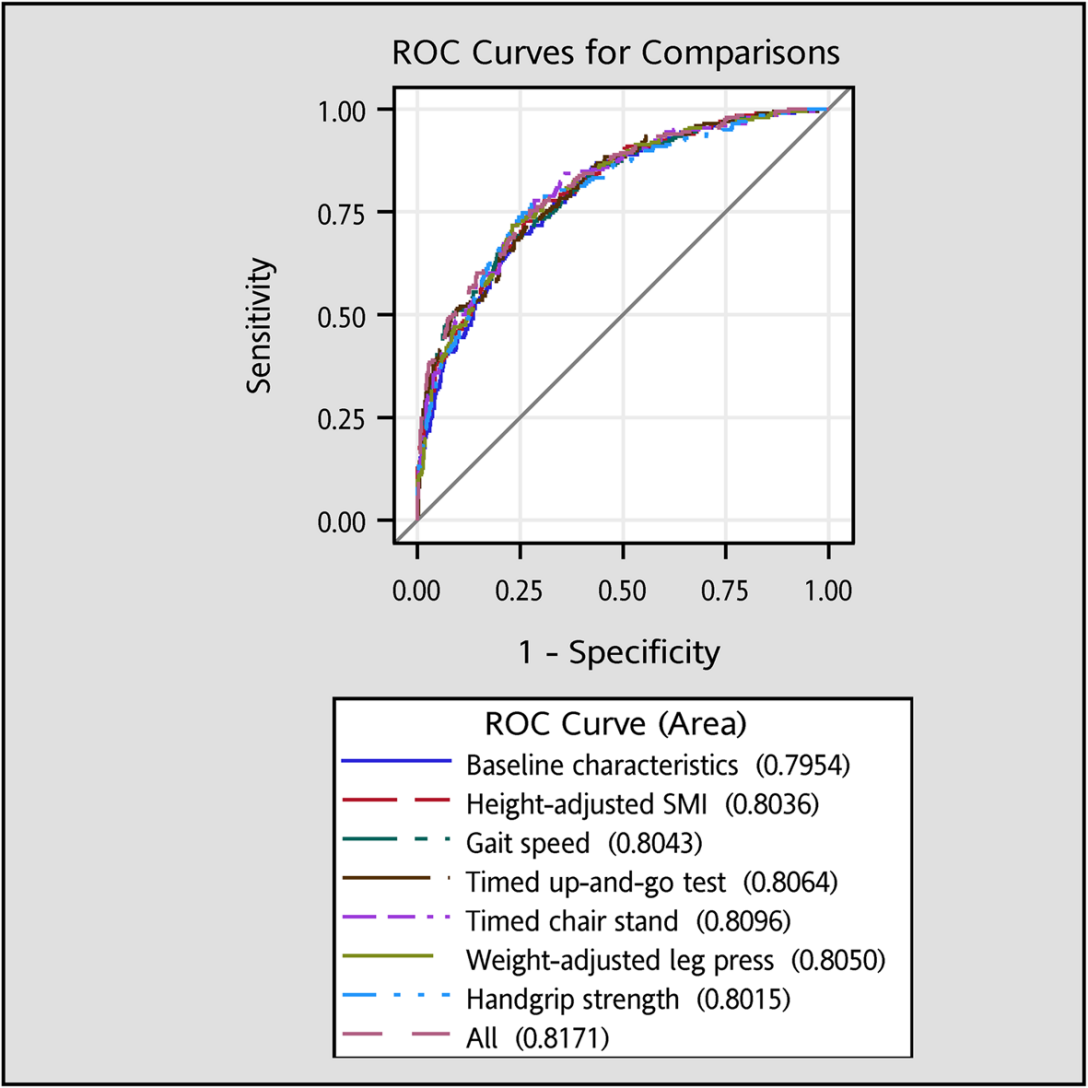
The areas under the receiver operating characteristic curves for all-cause mortality. The AUCs for models with additional consideration of TUG and all skeletal muscle mass and physical performance variables were significantly higher than that for model with baseline characteristics (*p* < 0.01)

Then the discriminatory ability was assessed by the AUROCs of baseline characteristics, hSMI, and physical performances for predicting mortality (Fig. 1), and the results indicate these skeletal muscle index and physical performance measures had good predictive power (all area under the curve (AUC) > 0.80). The values of AUCs for models with additional consideration for TUG and all skeletal muscle index and physical performance variables were significantly higher than that for model with baseline characteristics (both *p* < 0.01).

**Fig. 2:**
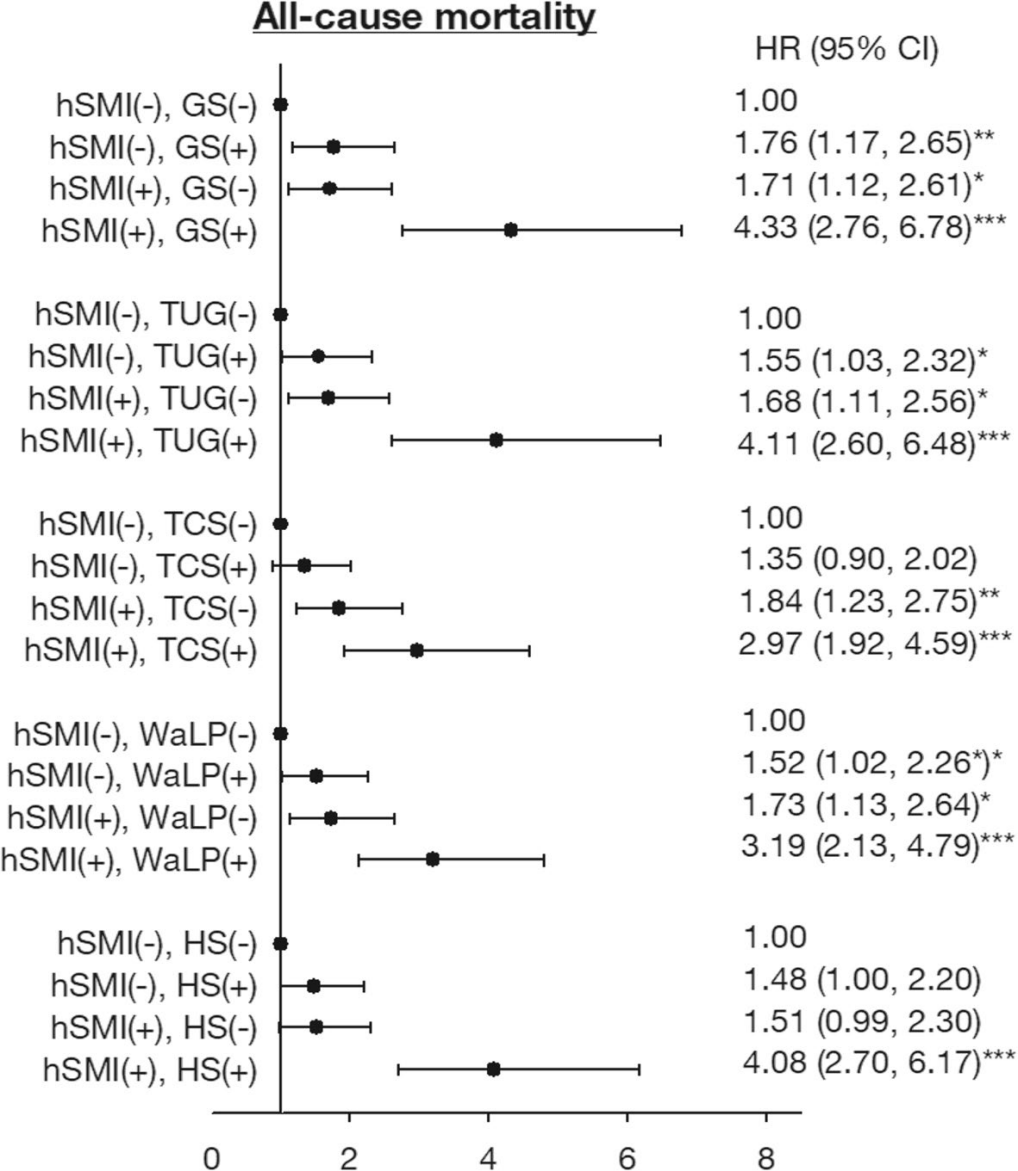
Joint relationship of height-adjusted SMI with gait speed, TUG, TCS and WaLP on risk for all-cause mortality. Adjusted for age, sex, education, marital status, smoking, alcohol drinking, regular exercise, exercising program, hypertension, diabetes mellitus, heart disease, stroke, cancer, cognitive impairment, fasting plasma glucose, triglyceride, high-density lipoprotein, low-density lipoprotein, and estimated glomerular filtration rate. + : the highest HR group; -: the other group

The interactions between skeletal muscle index and physical performance were assessed and none of these interactions are significant (all *p* > 0.05). Thus, the joint effects of hSMI with GS, TUG, TCS, or HS on death risks were displayed (Fig. 2). The results showed that the lowest hSMI quartile combined with the lowest GS quartile, highest TUS quartile, highest TCS quartile, lowest WaLP, or lowest HS were associated with three- to fourfold higher mortality (HRs = 4.33, 95% CI: 2.76–6.78; 4.11, 2.60–6.48, 2.97, 1.92–4.59; 3.19, 2.13–4.79; 4.08, 2.70–6.17, respectively).

## Discussion

In this 12-year follow-up prospective study, hSMI and physical performance measures of GS, TUG, TCS, WaLP, and HS exhibited independent association with long-term mortality among older adults. A positive linear association was also observed with the numbers of risk factors. Among the measures of skeletal muscle index and physical performance, the additional consideration of TUG or all measures had significantly improved the discriminatory ability for all-cause mortality.

A previous meta-analysis revealed an inverse association between hSMI and mortality in older adults, and the study’s results showed a smaller magnitude of difference in muscle mass between older adults who died and survived [44]. Although the present study used different perspective to examine the association between hSMI and mortality, we observed that older persons with higher levels of hSMI were associated with a lower risk of 12-year mortality. Our study’s findings were consistent with those reported in a Korean elderly cohort with a 5-year follow-up period, indicating that decrease in appendicular skeletal mass was associated with mortality in both sexes [28]. In addition, two observational cohort studies in Asia conducted in special populations reported that hSMI was associated with mortality in women among nonagenarians and centenarians in China [10] or patients with type 2 diabetes in Japan [45]. On the contrary, in a study conducted in Japanese persons aged between 40–79 years old of the Longitudinal Study of Aging, Japan, a higher level of hSMI was not associated with the composite outcome measure of mortality or disability after multivariate adjustment [13]. The possible explanation why muscle mass cannot be used to predict mortality or disability in this Japan study was that the study subjects included adults and elderly (age range of 40–79 years), and adults generally face a lower risk of mortality than elders and were less likely to suffer lean muscle loss. This can be supported by the lower overall cumulative mortality or disability (19.67%) in Japan study, compared with ours (30.89%).

In terms of physical performance, several studies have indicated that physical performance measures of GS, TUG, TCS, WaLP, and HS were better than the SMI in older adults for predicting future death [28] and can be used as a clinical screening tool [16–23]. Therefore, the combination of physical performance and SMI is likely to strengthen its association with adverse outcomes. Physical performance measures can be performed with relative ease in a clinical setting when evaluation of the physical function component of sarcopenia is required. Our study demonstrated that the combination of hSMI with physical function improved mortality risk prediction.

Sarcopenia is a complex syndrome that is characterized by progressive and generalized loss of muscle mass and strength or physical performance with an increased risk for mortality [46]. In 2010, the EWGSOP promulgated a sarcopenia definition that has been widely used either in clinical settings or community that advances in scientific research of sarcopenia [2]. This original criteria of diagnosis for sarcopenia is defined as low muscle mass plus low muscle strength or physical performance. Based on the accumulative scientific evidence, 2018 EWGSOP definition used low muscle strength as the primary parameter for defining probable sarcopenia; a confirmed sarcopenia diagnosis is made when probable sarcopenia with the presence of low muscle quantity or quality is detected [2]. When the presence of low muscle strength, low muscle quantity/quality and low physical performance are detected, sarcopenia is considered to be severe. The AWGS 2014 consensus defined sarcopenia similar to the 2010 EWGSOP definition [47]. Its revised definition in 2019 retains the previous definition due to limited data on Asian people [48]. Our study indicated that heighted-adjusted SMI and physical performance (GS, TUG, TCS, WaLP, and HS) are independent mortality predictors of traditional risk factors, consistent with previous studies [10–28, 45, 49]. Our study’s findings support the use of heighted adjusted SMI in AWGS sarcopenia definition because heighted adjusted SMI was the only muscle mass index associated with mortality. In addition, our study’s findings indicate men with handgrip strength lower than 28.69 kg and women lower than 18.79 kg, determined by first quartile, were associated with an increased risk of mortality, which is in accordance with the cutoff point of handgrip strength in AWGS sarcopenia definition (28 kg for men and 18 kg for women).

This present study is a prospective, community‐based observational cohort research. Study data were collected using standardized procedures and measurement instruments to ensure the validity and reliability. Death records of study samples were obtained through the linkage of national death dataset of Taiwan Ministry of Health and Welfare and have been regularly assessed for validity. Several limitations should be mentioned when interpreting the results of this study. First, the SMI measured by using DXA may be inaccurate in the calculation of the overall muscle mass throughout the body; however, DXA is a reliable method for use as a reference standard (but not a gold standard) for measuring muscle mass [50]. Second, our study covered the noninstitutionalized older adults, omitting residents in the nursing home who have high risks of dementia and death, which may limit the generalization of our study’s findings. The possibility of selection bias may occur during identification of the study population. However, based on the similar distributions of age and gender between our sample and target population, the potential selection bias may be minimized. Our findings can be generalized to older population in Taichung city.

## Conclusion

The hSMI is a useful indicator in the prediction of long-term mortality in men but not in women. Consistently, physical performance measures of walking speed, TUG, TCS, waLP, and HS are also considered valid or meaningful predictors. Our study suggested that hSMI and physical performance are key indicators, individually or in combination, in the prediction of mortality, which may help target interventions to improve survival among older adults.

## Supplementary Information

**Additional file 1:** **Supplement Figure S1. **The flowchart of recruitment procedures of the current study. **Supplement Figure S2**. Kaplan-​Meier curves for all-cause mortality by quartiles of (a) height-adjusted SMI, (b)gait speed, (c)TUG, (d)TCS, (e)WaLP, and (f) handgrip strength.

## Data Availability

The datasets generated and/or analyzed during the current study are not publicly available due to the policy declared by National Health Insurance in Taiwan but are available from the corresponding author on reasonable request.

## Abbreviations

SMI: Skeletal muscle index
TUG: Timed up-and-go
EWGSOP: European Working Group on Sarcopenia in Older People
AWGS: Asian Working Group for Sarcopenia
GS: Gait speed
HRC: Human Research Committee
MMSE: Mini-mental State Examination
DXA: Dual-energy X-ray absorptiometry
hSMI: Height-adjusted skeletal muscle index
wSMI: Weight-adjusted skeletal muscle index
TCS: Timed chair stands
waLP: Weight-adjusted leg press
HS: Handgrip strength
SD: Standard deviation
HR: Hazard ratio
CIs: Confidence interval
AUROCs: Areas under the receiver operating characteristic curves
AUC: Area under the curve
