# Supplementary material for: Independent and joint associations of skeletal muscle mass and physical performance with all-cause mortality among older adults: a 12-year prospective cohort study
Source: BMC Geriatr. 2022 Jul 18;22:597. doi: 10.1186/s12877-022-03292-0 (PMC9295364; doi:10.1186/s12877-022-03292-0)
Supplement: Supplementary file 1 — Additional file 1: Supplement Figure S1. The flowchart of recruitment procedures of the current study. Supplement Figure S2. Kaplan-​Meier curves for all-cause mortality by quartiles of (a) height-adjusted SMI, (b)gait speed, (c)TUG, (d)TCS, (e)WaLP, and (f) handgrip strength. [file 12877_2022_3292_MOESM1_ESM.docx]

573 refused to undertake the dual-energy X-ray absorptiometry examination

133 lack of information on baseline characteristics or physical performance

2750 eligible subjects

Eight administrative neighborhoods units in Taichung community

3997 individuals with age ≥ 65 years old

1247 excluded

122 deaths

124 errors of the registry

52 institutionalization

949 moving out of the area

886 refused to participate

490 not met at home during three visits made by the interviewers

27 hospitalization

1347 elders agreed to participate

(response rate = 49.0%)

641 complete data for analysis

**Supplement Figure S1.** The flowchart of recruitment procedures of the current study

| (a)  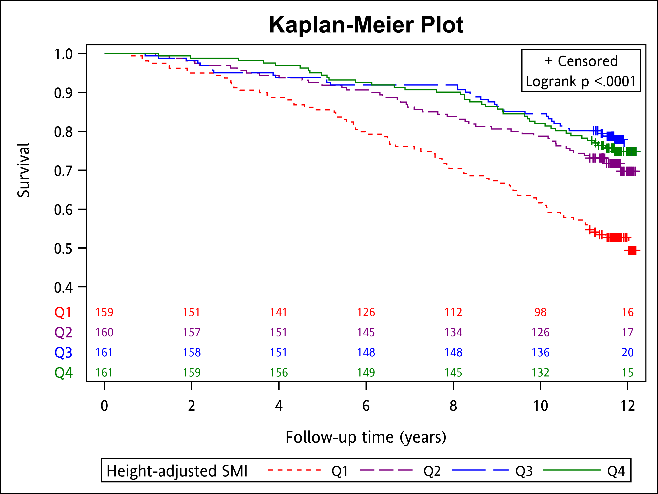 | (b)  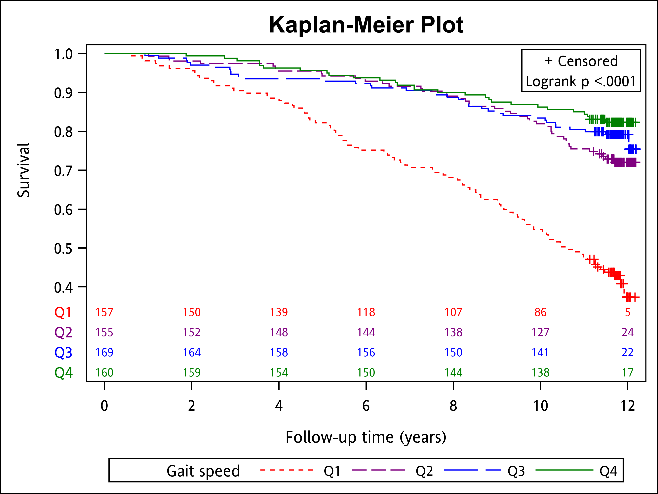 | (c)  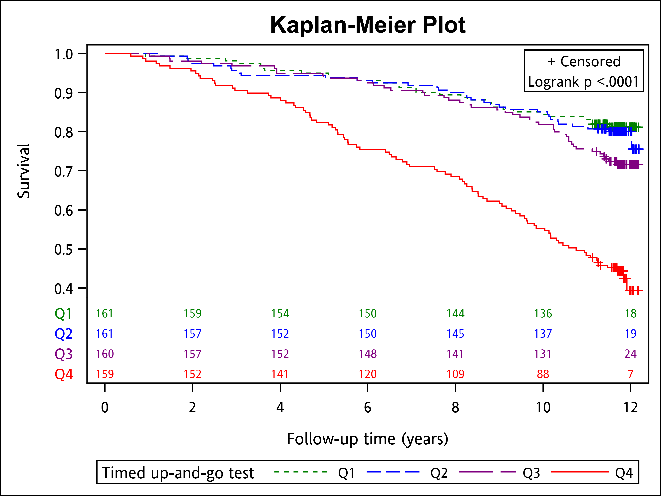 |
| --- | --- | --- |
| (d)  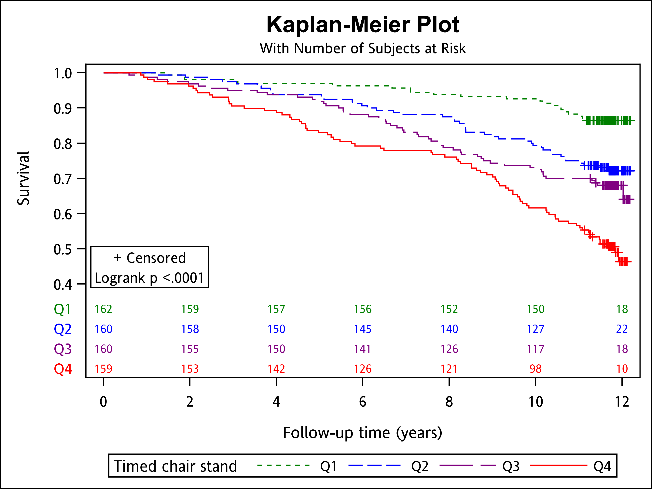 | (e)  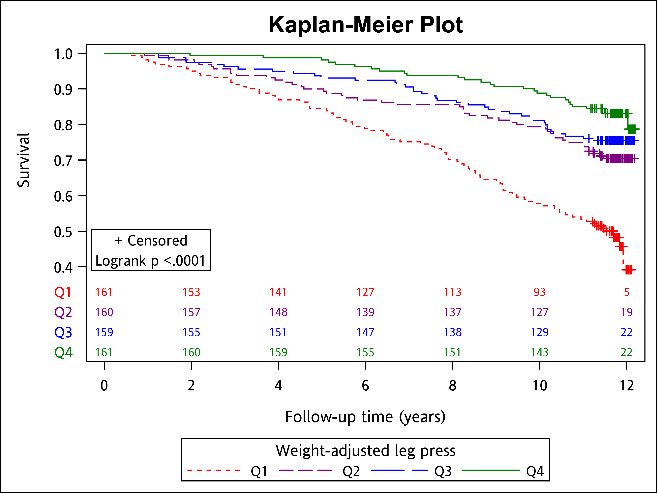 | (f)  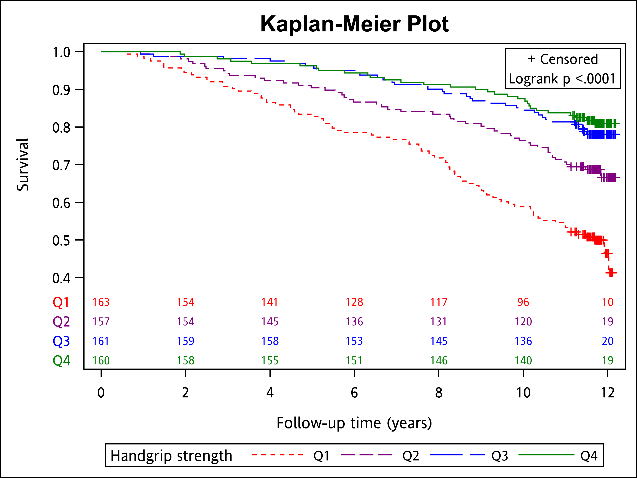 |

**Supplement Figure S2**. Kaplan-​Meier curves for all-cause mortality by quartiles of (a) height-adjusted SMI, (b)gait speed, (c)TUG, (d)TCS, (e)WaLP, and (f) handgrip strength
